# Supplementary material for: Relative Contribution of Blood Pressure and Renal Sympathetic Nerve Activity to Proximal Tubular Sodium Reabsorption via NHE3 Activity
Source: Int J Mol Sci. 2022 Dec 26;24(1):349. doi: 10.3390/ijms24010349 (PMC9820392; doi:10.3390/ijms24010349)

Figure S1. Protocol 1—Experiment timeline—sham vs. Bicuculline (Bic) paraventricular nucleus of the hypothalamus (PVN) (pre vs. post).

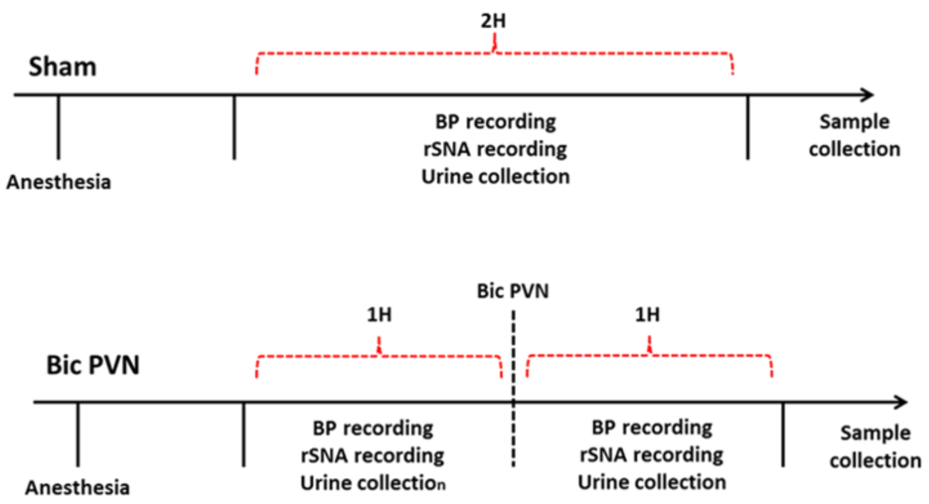


Figure S2. Protocol 2: Experiment timeline—Baseline × RNS × RNS+ Bicuculline (Bic) paraventric-ular nucleus of the hypothalamus (Bic PVN).

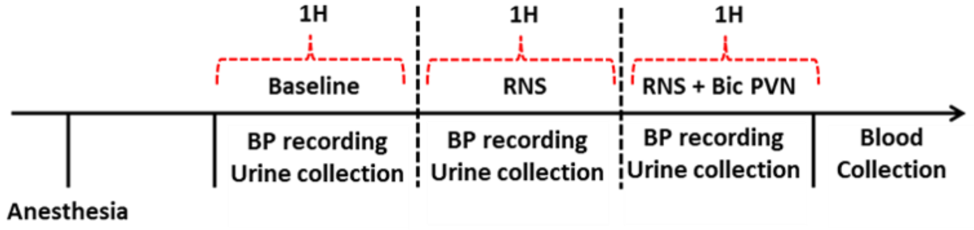

Supplement: Supplementary file 1 [file ijms-24-00349-s001.zip › ijms-2075122-supplementary.pdf]
